# Prevalence of mental disorders among middle-aged population of primary healthcare centers in Northeastern Iran

**DOI:** 10.1186/s12889-023-17598-0

**Published:** 2024-01-03

**Authors:** Mehdi Talebi, Ali Taghipour, Amene Raouf-Rahmati, Ehsan Musa Farokhani, Saeed Ghaffariyan-Jam, Azadeh Samarghandi, Maryam Nemati, Ahmad Nemati

**Affiliations:** 1 Department of Community and Family Medicine, Faculty of Medicine, Mashhad University of Medical Sciences, Mashhad, Iran; 2 Health Sciences Research Center, Department of Biostatistics and Epidemiology, School of Health, Mashhad University of Medical Sciences, Mashhad, Iran; 3 Department of Parasitology and Mycology, School of Medicine, Mashhad University of Medical Sciences, Mashhad, Iran; 4 Epidemiology, Mashhad University of Medial Sciences, Mashhad, Iran; 5 Family Medicine, Mashhad University of Medical Sciences, Mashhad, Iran; 6 Department of Psychology, Faculty of Education Sciences and Psychology, Ferdowsi University of Mashhad, Mashhad, Iran; 7 Internal Medicine, Endocrinology and Diabetes Optum, Laguna Niguel, Ca USA; 8 Mashhad University of Medical Sciences, Mashhad, Iran

**Keywords:** Primary Health Care, Mental disorders, Middle aged, Iran

## Abstract

**Background:**

Primary healthcare centers (PHCs) serve as the cornerstone of accessible medical services in society, playing a crucial role in screening, detecting, and treating various health issues. This study aimed to investigate the prevalence of psychiatric disorders in middle-aged individuals who refer to PHCs and the potential of PHCs in diagnosing mental disorders.

**Methods:**

This cross-sectional study was implemented at PHCs under the supervision of Mashhad University of Medical Sciences (MUMS) in northeast Iran in 2018. The enrolled subjects were middle-aged adults who had electronic medical records in SINA, an integrated health management system, and the electronic medical records of MUMS. The prevalence of psychiatric disorders by type and their relationship with demographic information was evaluated by a Chi-square test using SPSS 22.

**Results:**

This study involved 218,341 middle-aged participants. Prevalence of psychiatric disorders was 8.59%, and depression (53.72%) and anxiety (42.02%) were the most common psychiatric disorders in both males and females. The prevalence of mental disorders was significantly higher in females than in males (88.18% vs. 18.81%; *P* < 0.0001). Indeed, a significant higher prevalence of depression, anxiety, somatoform, childhood psychiatric disorder, and bipolar disorders was observed in females compared to males (*P* < 0.05). In addition, individuals between the age of 45–60 years, and those from rural areas showed more prevalence of mental disorders than others, but these differences were not significant.

**Conclusions:**

Considering the previous studies in Iran, the prevalence of mental disorders among patients presenting to PHCs was noticeably lower than expected rates. It seems probable that this huge difference is due to poor screening and detection of mental illness in PHCs of MUMS. It is recommended that health policymakers pursue specific measures to make PHCs more helpful for people with mental health problems in the community.

## Introduction

Mental disorder is a prevalent medical condition that significantly impacts the quality of life across communities [1]. Mental illnesses have been identified as one of the most significant reasons for global burden diseases (GBDs) [2]. It has been previously noted that mental disorders are associated with a decline in social functioning, decreased work productivity, and disturbances in familial relationships; thereby leading to a rise in healthcare and economic expenditures [3]. Moreover, the world economy suffered 1 trillion dollars detriment in 2020 due to the high prevalence of mental illnesses such as depression and anxiety [4]. World Health Organization (WHO) announced that in 2001 approximately 400 million people around the world suffered from mental or neurological disorders or from psychosocial problems such as those related to alcohol and drug abuse [5] while, in 2019, it upward to 970 million individuals worldwide based on WHO reports [6]. Epidemiological data from community studies conducted in European countries in 2005 demonstrated that about 27% of the adult EU population, 18–65 of age, have at least one psychological disorder in a 12-month period [7]. In a survey study by the WHO World Mental Health conducted in 2006, the 12-month prevalence of various psychiatric disorders in America is estimated at 27% [8]. In line with European countries, the Eastern Mediterranean region is facing a growing incidence of chronic diseases, including psychiatric diseases [9]. In the first study on the burden of psychiatric diseases in the Eastern Mediterranean region, psychiatric diseases accounted for 5.6% of the total burden of diseases [9]. The burden of mental disorders in the Eastern Mediterranean region, in 2015 was estimated higher than global levels, particularly for women [10]. A high rate (46.3%) of mental disorders was found in 2,948 primary care consecutive attendees from six primary care clinics in Israel, West of Asia compared to other countries [11]. In line with other parts of the world, Iran has a high mental disorder burden. A nationwide investigation in Iran remarked that the 2015 prevalence of psychiatric disorders was 23.4% [12]. Based on a recent systematic review, mental illnesses have become a significant national issue and we are facing a growing trend in Iran [13].

Assessment of mental health conditions requires preventive measures, sensitive screening protocols, and diagnosis accompanied by suitable treatment and follow-up [14]. Primary Healthcare Centers (PHCs) are considered the first line of communication between the population and the healthcare system [15]. PHCs provide health services equitably and cost-effectively for social health and welfare enhancement [16]. In Iran, PHCs are responsible for providing a wide range of essential health services including screening, prevention, treatment, mental health training, and referral to higher-level healthcare systems if necessary. University of Medical Sciences owns a main health center and supervises all secondary health centers in urban and margin areas. Healthcare services in some provinces are managed by more than one University of Medical Sciences. Although there are five medical universities in Khorasan-e-Razavi province, the majority of the population is under the coverage of Mashhad University of Medical Sciences (MUMS). In other words, the non-marginal and marginal health centers of Mashhad, Kashmar, Quchan, Torbat-e Jam, Taybad, Chenaran, and Sarakhs are under the coverage of MUMS. PHC members include general physicians, nurses, psychiatrists, midwives, and other health workers [17]. In Iran, mental health services can be accessed through diverse facilities like psychiatric hospitals and private clinics. However, for more than 40 years since mental health services were integrated into PHCs, PHCs have become a major source of easily accessible and affordable mental health care for all residents [18]. So far, this method has shown remarkable benefits by improving health outcomes and increasing access to healthcare services [19, 20]. The integration of mental health services into PHCs has demonstrated valuable achievements in other countries [21]. Based on some research conducted in primary care settings in Spain and Saudi Arabia, 30% and 28.5% of the population suffered from some degree of psychiatric disorders respectively [22, 23]. Previous studies in Iran assessed psychological distress and the incidence of mental illnesses. Tabrizi et al. concluded that the primary healthcare system in Iran needs reforms due to changes in common disease patterns and socio-economic structures [24]. Initial reports remarked that the integration of mental health services into PHCs is a successful approach to improving mental healthcare in Iran [25]. On the other hand, recent evidence highlights the importance of reforms to overcome some barriers to mental health care in primary care settings to be more qualified to diagnose, treat, refer, and follow up with people with mental illness, as well as prevent mental illnesses [26]. Hitherto, the prevalence of mental disorders among individuals referring to PHCs has not been investigated in Iran.

This study aims to investigate the prevalence of mental illnesses among middle-aged individuals referring to PHCs of Mashhad University of Medical Sciences and discuss the probable defects of PHCs in the screening and early diagnosis of patients with mental disorders.

## Method

This analytical cross-sectional study was carried out in 2018 using data from SINA. Ethical approval was provided by the Ethics Committee of the Research Department of Mashhad University of Medical Sciences IR.MUMS.MEDICAL.REC. 1397. 144.

### Participants

Middle-aged individuals, as defined by national health guidelines in Iran (ages 30 to 60), were included in the study [27]. In this study, individuals of middle age who had been registered in SINA during the designated period were selected for inclusion.

### Procedure

Over a year, a total of 218, 341 middle-aged individuals were registered in electronic mental health records in SINA system. SINA is an integrated hospital management system and electronic health record software that was implemented in June 2015 at all levels of the Health Deputy by MUMS in order to modify the health system infrastructure and provide a high-quality treatment process and electronic services to the community [28, 29]. SINA system displays the participants underlined diagnosed mental illness and also conducts mental illness screening. In PHCs of MUMS, the Kessler and colleagues (K6) scale in the Persian language is employed as a six-item questionnaire designed to evaluate an individual’s emotional well-being and serve as a screening tool for potential mental disorders [30]. Kessler’s Psychological Distress Scale for identifying mental disorders in the general population was compiled by Kessler and his colleagues in 2002 in two forms of 6 questions (K6) and 10 questions (K10) and has been used in various studies due to its high sensitivity. The questions are taken from among 18 commonly used screening scales, and based on the most modern response theory methods, it is determined which of these questions has the best ability to screen psychological distress during the last month [31]. Only some questions were selected for this test that were consistent with socio-demographic intensity [31]. K6 has not been used in Persian until 2015. In 2015, Dadfar et al. examined and confirmed the reliability and validity of the Persian form of the K6 in 103 outpatients with psychological problems. The Cronbach’s alpha coefficient of the Persian form of the K6 was 0.885. The intercorrelations between the items ranged from 0.154 to 0.805, and the item-total correlations ranged from 0.503 to 0.923. This study concluded that the test-retest reliability was satisfactory (r = 0.80, *P* < 0.001). One factor was identified in exploratory factor analysis with eigenvalues 3.875 and % of variance 64.577%) labeled: Psychological Distress. The results provide preliminary support for the reliability and validity of the K6 in Iranian clinical and non-clinical care services [32]. Moreover, in a study with the aim of investigating the factor structure, convergent and criterion validity gender invariance of K6 among Bojnourd residents, it displaced that the structure of an agent explains 58.18% of the variance. The internal consistency of the scale was confirmed by Cronbach’s alpha of 0.86 and split-half, 0.83. This study concluded that K6 have good validity and reliability in the sample of men and women aged between 18 and 30 and can be used as a precise instrument in this regard [33]. The answers are in Likert form and include: never, rarely, sometimes, most of the time, and always. Scores range from 0 for never to 4 for always [32]. The questions of this screening scale, which is included in the SINA system, are described in Table 1 [32].

**Table 1:** The questions of this screening scale, which is included in the SINA system of MUMS

Number	Question
1	During the last 30 days, about how often did you feel nervous?
2	During the last 30 days, about how often did you feel restless or fidgety?
3	During the last 30 days, about how often did you feel that everything was an effort?
4	During the last 30 days, about how often did you feel so sad that nothing could cheer you up?
5	During the last 30 days, about how often did you feel hopeless?
6	During the last 30 days, about how often did you feel worthless?

Participants who achieved a superior score on the K6 test, utilizing a standard cutoff, received a personal visit from the general practitioner of PHCs. If individuals had a mental disorder, the appropriate treatment was started by a general practitioner or patients were referred to a higher-level referral system such as community mental health centers (CMHCs), where a consultation with a psychiatrist can take place [34]. Henceforth, the type of psychological disorders was diagnosed by either a general practitioner or a psychiatrist and subsequently was recorded in the mental health electronic database. Data were collected using demographic information including gender, age, and area of residence (urban or rural). Furthermore, the specific type of mental disorder was obtained and was categorized based on DSM-IV Axis I.

### Data Analysis

The names were converted to the codes and were entered into SPSS 22 (Armonk, NY: IBM Corp Armonk, NY: IBM Corp). The Chi-squared test was used to compare the frequency of mental illnesses between the two groups (male vs. females, age < 45 vs. >45 years, urban residents vs. rural residents). The level of statistical significance was determined as a *P*-value less than 0.05.

## Results

In this study, 218,341 middle-aged participants based on data from the electronic mental health records of SINA system were enrolled. The mean age was 43.05 ± 9.24 years old and the majority (71.6%) were women. Moreover, the participants aged between 30 and 45 years old and those from urban areas were more predominant than the other groups. Approximately, one of twelve participants (8.59%) showed some level of mental disorders, while 91.4% did not. More detail in Table 2.

**Table 2:** Demographic characteristics and psychological status of middle-aged patients referring to primary healthcare centers of Khorasan-e-Razavi, 2018

Variables	Number	Frequency (%)
**Total**	218,341	100
**Gender** Male Female	61,933 156,408	28.4 71.6
**Age** 30–45 years old 46–60 years old	127,588 90,753	58.4 41.6
**Area of residence** Urban areas Rural areas **Psychological status**	137,850 80,491	63.1 36.9
Normal Abnormal	199,578 18,763	91.4 8.59

The frequency of psychiatric disorders is set out in Table 3. Depression (53.72%) and anxiety (42.02%) were the most common mental disorders in both males and females. In addition, 16 patients (0.08%) showed some mental health issues that have been initiated in childhood and lasted into adulthood such as attention deficit hyperactivity disorder (ADHD). As shown in Table 4, the prevalence of mental disorders was significantly higher in females than in males (88.18% vs. 18.81%; *P* < 0.0001). Indeed, a significant higher prevalence of depression, anxiety, somatoform, childhood psychiatric disorder, and bipolar disorders was observed in females compared to males (*P* < 0.05). In addition, individuals between the age of 45–60 years, and those from marginal areas showed more prevalence of mental disorders than others, but these differences were not significant.

**Table 3:** Prevalence of mental disorders among middle-aged patients referring to primary healthcare centers of Khorasan-e-Razavi, 2018

Psychological status	Total	Sex		Age		Area of residence	
		Males; n (%)	Females; n (%)	30–45 years old; n (%)	45–60 years old; n (%)	Urban areas; n (%)	Rural areas; n (%)
Psychotic disorders	206 (1.09)	108 (4.87)	98 (0.59)	88 (1.04)	118 (1.14)	41 (0.44)	165 (1.74)
Bipolar and related disorders	298 (1.58)	93 (4.19)	205 (1.23)	137 (1.61)	161 (1.56)	142 (1.52)	156 (1.64)
Anxiety and related disorders	7611 (40.56)	736 (33.19)	6875 (41.55)	3538 (41.81)	4073 (39.53)	4120 (44.33)	3491 (36.86)
Depressive disorders	9730 (51.85)	910 (41.04)	8820 (53.3)	4283 (50.62)	5447 (52.87)	4735 (50.95)	4995 (52.74)
Somatoform disorders	890 (4.74)	359 (16.19)	531 (3.2)	405 (4.78)	485 (4.7)	248 (2.66)	642 (6.77)
Neurocognitive disorders	12 (0.06)	6 (0.27)	6 (0.03)	0 (0)	12 (0.11)	2 (0.02)	10 (0.1)
Childhood psychiatric disorder persist into adulthood	16 (0.08)	5 (0.22)	11 (0.06)	10 (0.11)	6 (0.05)	5 (0.05)	11 (0.11)
**Total patients**	18,763 (100)	2217 (100)	16,546 (100)	8461 (100)	10,302 (100)	9293 (100)	9470 (100)

*Chi-square test. n, number

**Table 4:** Comparison of the prevalence of mental disorders among middle-aged people referring to primary healthcare centers of Khorasan-e-Razavi based on demographic characteristics, 2018

Psychological status	Total	Sex			Age			Area of residence		
		Males; n (%)	Females; n (%)	*P*-value	30–45 years old; n (%)	45–60 years old; n (%)	*P*-value	Urban areas; n (%)	Rural areas; n (%)	*P*-value
Psychotic disorders	206 (100)	108 (52.42)	98 (47.57)	0.08	88 (42.71)	118 (57.28)	**< 0.05**	41 (19.9)	165 (80.09)	**< 0.0001**
Bipolar and related disorders	298 (100)	93 (31.2)	205 (68.79)	**< 0.0001**	137 (45.97)	161 (54.02)	0.07	142 (47.65)	156 (52.34)	0.08
Anxiety and related disorders	7611 (100)	736 (9.67)	6875 (90.32)	**< 0.0001**	3538 (46.48)	4073 (53.51)	0.07	4120 (54.13)	3491 (45.86)	0.07
Depressive disorders	9730 (100)	910 (9.35)	8820 (90.64)	**< 0.0001**	4283 (44.01)	5447 (55.98)	0.07	4736 (48.67)	4996 (51.34)	0.08
Somatoform disorders	890 (100)	359 (40.33)	531 (59.66)	**< 0.05**	405 (45.5)	485 (54.59)	0.07	248 (27.86)	642 (72.13)	**< 0.0001**
Neurocognitive disorders	12 (100)	6 (50)	6 (50)	0.09	0 (0)	12 (100)	**< 0.0001**	2 (16.66)	10 (83.33)	**< 0.0001**
Childhood psychiatric disorder persist into adulthood	16 (100)	5 (31.25)	11 (68.75)	**< 0.0001**	10 (62.5)	6 (37.5)	**< 0.0001**	5 (31.25)	11 (68.75)	**< 0.0001**
**Total patients**	18,763 (100)	2217 (11.81)	16,546 (88.18)	**< 0.0001**	8461 (45.09)	10,302 (54.9)	0.07	9293 (49.52)	9470 (50.47)	0.09

*Chi-square test. n, number

## Discussion

The study aimed to explore the prevalence of mental disorders among middle-aged individuals receiving PHC services under the supervision of Mashhad University of Medical Sciences. Findings revealed that only 8.59% of participants exhibited varying degrees of mental illnesses, with 11.81% among males and 88.18% among females. The results indicated a significantly higher prevalence of mental illness in females than in males (*P* < 0.0001). Additionally, the prevalence of mental disorders was higher in the age group of 45 to 60 years and among rural residents compared to the age group of 30 to 45 years and urban residents, though these differences were not statistically significant.

This outcome is somewhat surprising considering the higher prevalence of mental illnesses among the general population in other research. In a K6 screening test among adults aged 18–65 under the health deputy of MUMS, 10.1% of participants (12.8% of females and 5.5% of males) experienced psychological distress [35]. The aforementioned study, however, reported the prevalence of psychological distress in patients of the same age as ours at 13.23% (30–65 years). The major difference between our study and the work done by Rahimi et al. is that, the authors investigated psychological distress that is more prevalent than mental disorders. It should be emphasized that psychological distress and mental illnesses are two separate situations. Individuals suffering psychological distress exhibit symptoms such as anxiousness and low mood and such individuals, but not all of them, have a high potential to develop mental illnesses [36]. However, there is some evidence of a higher prevalence of mental disorders in the general population. According to a comprehensive national survey conducted in Khorasan-e-Razavi province, the prevalence of mental disorders among the general population is estimated at 23.7%, with a higher proportion of females compared to males (26.9% vs. 20. 6%) [37]. Our study showed heterogeneous results compared to the mentioned study which was carried out in the same region. A cohort study estimated the 37.1% prevalence of mental illness among the citizens of Tehran [38]. A most recent study by Fakhari et al., conducted among 1000 aging people randomly selected from a general population of northwest Iran, revealed that 38.5% of the individuals showed one or more mental disorders (27.3% men vs. 47.2% women) [39]. These findings showed an apparent difference regarding the prevalence of mental disorders in the general population and populations of PHC services. Another study in the capital of Iran estimated the incidence of mental illnesses at 15.9% per 1000 populations in ten years after conducting and stabilizing integrated mental health in primary health care [40]. There are several studies worldwide indicate the prevalence of mental disorders among individuals attending PHC facilities. Based on an international survey in 18 countries, 24% of adult patients attending PHCs showed some level of mental disorders [41]. Some recent research has found higher rates of mental illness in different countries such as Brazil (41%), Qatar (26.7%), and Tanzania (24%) [42–44]. According to our findings and other similar findings worldwide, PHCs in Iran estimated a lower prevalence of mental illness than in other countries. The main reasons were discussed at the end. Our findings support previous studies regarding the higher rates of depression, anxiety disorders, and somatoform disorders compared to other mental illnesses in Iran [12, 45]. According to the study by Bahrami et al., individuals over the age of 15 in Tehran, showed depression, anxiety, and somatizations as the most prevalent psychiatric disorders [38]. In another work, the prevalence of major depressive disorder and any anxiety disorders was 16.6% and 16.7% respectively [39].

The current results demonstrated a significantly higher proportion of mental illness in females than in males. These outcomes are similar to those reported by Noorbala et al., [37] and Fakhari et al., among the general population [39]. Women may be more vulnerable to mental illness due to some biological factors, more environmental stressors, and a lack of social support. Notwithstanding, some psychiatric disorders, such as antisocial personality disorder and substance abuse are more prevalent in men [46]. Some research discussed gender differences in psychiatric disorders [47, 48].

This investigation focused on middle-aged patients, however, the prevalence of mental disorders in the age group of 45 to 60 years was higher than in the age group of 30 to 45 years (*P* < 0.0001). In concurrence with this finding, the study by Rahimi et al. observed a higher prevalence of psychological distress with the increasing age (7.4% in the age 18–29 years, 10.58% in the age 30–49 years, and 14.08% in the age 50–65 years) [35]. But, these results are in contrast to some studies showing that age between 25 and 45 has a higher risk for mental illnesses [49, 50].

Despite previous controversial results [51, 52], our study found that residents of rural areas showed a slightly higher prevalence of mental illnesses than those in urban areas. In line with our findings, a study reported a prevalence of any type of mental disorder at 55.3% for marginalized populations and 31.5% for non-marginalized populations [39]. For residents in rural areas, PHCs may be their primary healthcare option, potentially contributing to the higher prevalence of mental illnesses among marginalized populations. Moreover, the increased prevalence of mental disorders in marginalized populations may be attributed to their low socio-economic status.

Taking this evidence into consideration, it seems that the prevalence of mental disorder among patients referring PHCs of MUMS are lower compared to the general population in this country and also the population of PHCs in other countries. PHCs are widely recognized as affordable and easy-access settings for providing health care services for society. Notably, Zyaambo et al., demonstrated that mentally ill patients tend to utilize PHC facilities to a greater extent than healthy individuals [53]. Moreover, some previous studies indicated that the prevalence of mental illnesses among patients attending PHC settings was similar to or more than the general population [23, 43]. Therefore, a higher proportion of mental illness was expected in PHCs than in the general population.

*There are four potential reasons behind the low prevalence of mental illnesses in PHC settings of MUMS*:

1. Some psychiatric patients may not seek appropriate treatment due to a lack of awareness of their illness, especially in complex and infrequent mental disorders, denial of their mental condition, or fear of the stigma [54, 55]. Diagnosed psychiatric disorders in PHCs are documented in electronic medical records, creating stigma for patients, leading them to abstain from referring to PHC settings. It is crucial for PHCs to identify individuals with mental illness who do not seek healthcare services. To achieve this, PHC members can make phone calls or conduct face-to-face interviews with inhabitants to assess their psychological status.
2. Patients with mental symptoms may seek treatment at private clinics or specialized psychiatric facilities instead of PHC settings. Some patients may prefer to be seen by a psychiatrist at private clinics rather than a general physician. This preference may be influenced by the perception that medics in psychiatric hospitals or private clinics are more skillful and experienced. Additionally, this preference may be due to concerns about registering patients’ information in PHCs and the associated stigma, as discussed in the previous Sect. 
3. Mental illness was more prevalent in rural areas, where PHCs are the main healthcare service providers, and residents in such areas have limited access to other healthcare options like private clinics. This could be considered the third reason, as patients in rural areas may need more healthcare, and there may be a lack of sufficient health centers in these deprived and marginalized areas. Not all patients with psychological disorders may be identified.
4. PHC staff or screening tools used in PHCs may lack the capability to detect and distinguish mental disorders properly. Farhoudian et al. found in a review of studies before 2007 that the prevalence of psychiatric disorders varied, with a range of 29.1% based on screening tools to 21.9% based on clinical interviews [56]. Another summary of studies from 2007 to 2018 showed the prevalence of mental illnesses at 31% according to screening tools and 25.4% according to clinical interviews [13]. According to the procedure at PHCs of MUMS, patients suspected of mental disorders are screened using both assessment tools and clinical interviews. The definitive diagnosis of mental illness is based on clinical interviews, and only these patients are registered in the SINA system. The main screening scale used in PHCs of Mashhad is K6. Hejabi et al. reported a sensitivity of 0.73 for the Persian K6 and concluded that this scale is valid and reliable for evaluating psychological distress [57]. However, another study introduced the Distress Questionnaire-5 (DQ5) scale as a more accurate tool for identifying mental disorders compared to the K6/K10 [58]. Stolk et al. noted the potential for changes in the sensitivity of K6/K10 with cultural diversity [59]. Additionally, the K6 may fail to capture individuals with more moderate mental distress [60]. Therefore, it is suggested that lower optimal cutoff scores may improve the sensitivity of the K6.

The major limitation of the study is that, although this research was conducted in 2018, the screening procedures for mental disorders currently in use in PHCs under the supervision of Mashhad University of Medical Sciences are still based on the old method, primarily using the K6 scale for primary screening.

## Conclusion

The key finding of this study is that the prevalence of mental illnesses among Primary Healthcare Centers (PHCs) in Khorasan-e-Razavi was lower than in the general population, contrasting with rates in other countries where this prevalence is similar or higher than that of the general population. The research underscores the need for a comprehensive investigation into the prevalence of mental illness among individuals seeking services from PHCs in other provinces of Iran. Deficiencies in screening and identifying mental disorders may be attributed to subpar screening instruments or insufficient expertise among PHC staff. Some patients with mental illness may not adhere to treatment in PHCs due to stigma, and others may seek treatment in alternative healthcare facilities instead of PHCs. While this study outlines possible reasons, further research is necessary to comprehend the exact barriers from the perspective of patients and PHC staff. The present study recommends that health policymakers implement beneficial reforms in PHCs in Iran, incorporating more sensitive screening scales than the K6, active screening for mental illness instead of conventional methods, and the retraining and enhancement of PHC staff experiences and skills.

## Data Availability

The data supporting this study’s findings are available from the corresponding author upon request.
